# Case report: An invasive and giant hemolymphangioma of the pancreas in a young man

**DOI:** 10.3389/fonc.2025.1524739

**Published:** 2025-02-13

**Authors:** Pin Wang, Mancai Wang, Yongyong Liu

**Affiliations:** Department of General Surgery, Second Hospital & Clinical Medicine School, Lanzhou University, Lanzhou, China

**Keywords:** pancreas, hemolymphangioma, surgery, cystic tumor, beaver tail liver

## Abstract

Hemolymphangioma is a rare benign tumor, with only 12 reported cases in the pancreas as of May 2024. We present an invasive and giant hemolymphangioma of the pancreas in a young man who experienced abdominal pain and left epigastric distension for approximately 10 days. Imaging studies revealed a large cystic tumor located in the body and tail of the pancreas, which was compressing the partial lesser curvature of the stomach and spleen, displaying a “beaver tail” liver appearance on computed tomography scans. After surgery, he was diagnosed with hemolymphangioma of the pancreas, and there were no signs of recurrence upon follow-up. We conclude that diagnosing hemolymphangioma of the pancreas can be challenging. Whenever possible, radical surgical resection should be performed, and long-term follow-up is essential.

## 1 Introduction

Hemolymphangioma is a mixed malformation involving lymphatic vessels and venules, primarily found in the head and neck (1), and less commonly in the pancreas. It is classified as a rare primary cystic tumor. According to the literature, hemolymphangiomas of the pancreas account for approximately 0.1% of all pancreatic tumors (2). The main symptoms and signs associated with hemolymphangioma include the presence of an abdominal mass and distension, which can be challenging to distinguish from other pancreatic tumors, leading to potential misdiagnosis. Most cases are identified through imaging examination and confirmed by pathological analysis. In this study, we report a case of an invasive and giant hemolymphangioma of the pancreas in a 24-year-old man.

## 2 Case report

**Figure 1:**
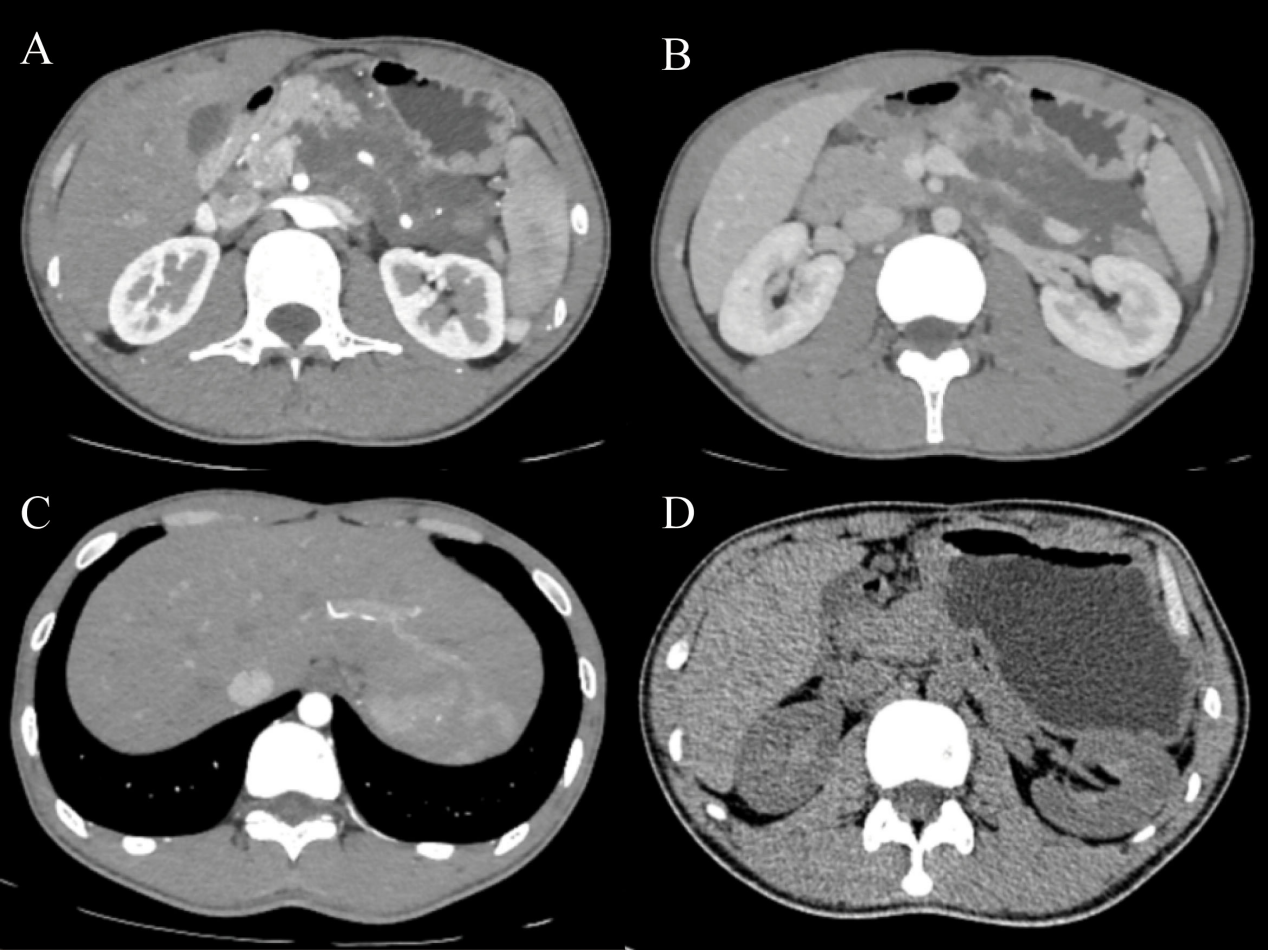
Abdominal CT demonstrates a multilocular cystic tumor in the body and tail of the pancreas, with septums noted within the cystic region **(A)**. The tumor surrounds the superior mesenteric artery and its branches, and the splenic artery and vein, with segmental narrowing of the splenic vein **(A, B)**. A “beaver tail” appearance of the liver was noted **(C)**. There were no signs of recurrence **(D)**.

A 24-year-old male patient was admitted to our hospital with abdominal pain and left epigastric distension that lasted approximately 10 days. Physical examination revealed deep tenderness in the left upper abdomen. Laboratory data, including renal and liver function tests, complete blood count, urine and stool routine examinations, and tumor markers such as CA19-9, CEA, and CA12-5 were all within normal ranges. The ultrasonography and computed tomography (CT) scan findings indicated a large cystic tumor located in the body and tail of the pancreas, which was compressing the partial lesser curvature of the stomach and spleen, with a diameter of 98 mm and attenuation values of 33-65 Hounsfield units. No enlarged lymph nodes were observed. Septums were noted within the cystic region. The tumor surrounded the superior mesenteric artery and its branches and the splenic artery and vein, with segmental narrowing of the splenic vein and a “beaver tail” appearance of the liver (**Figure 1**).

**Figure 2:**
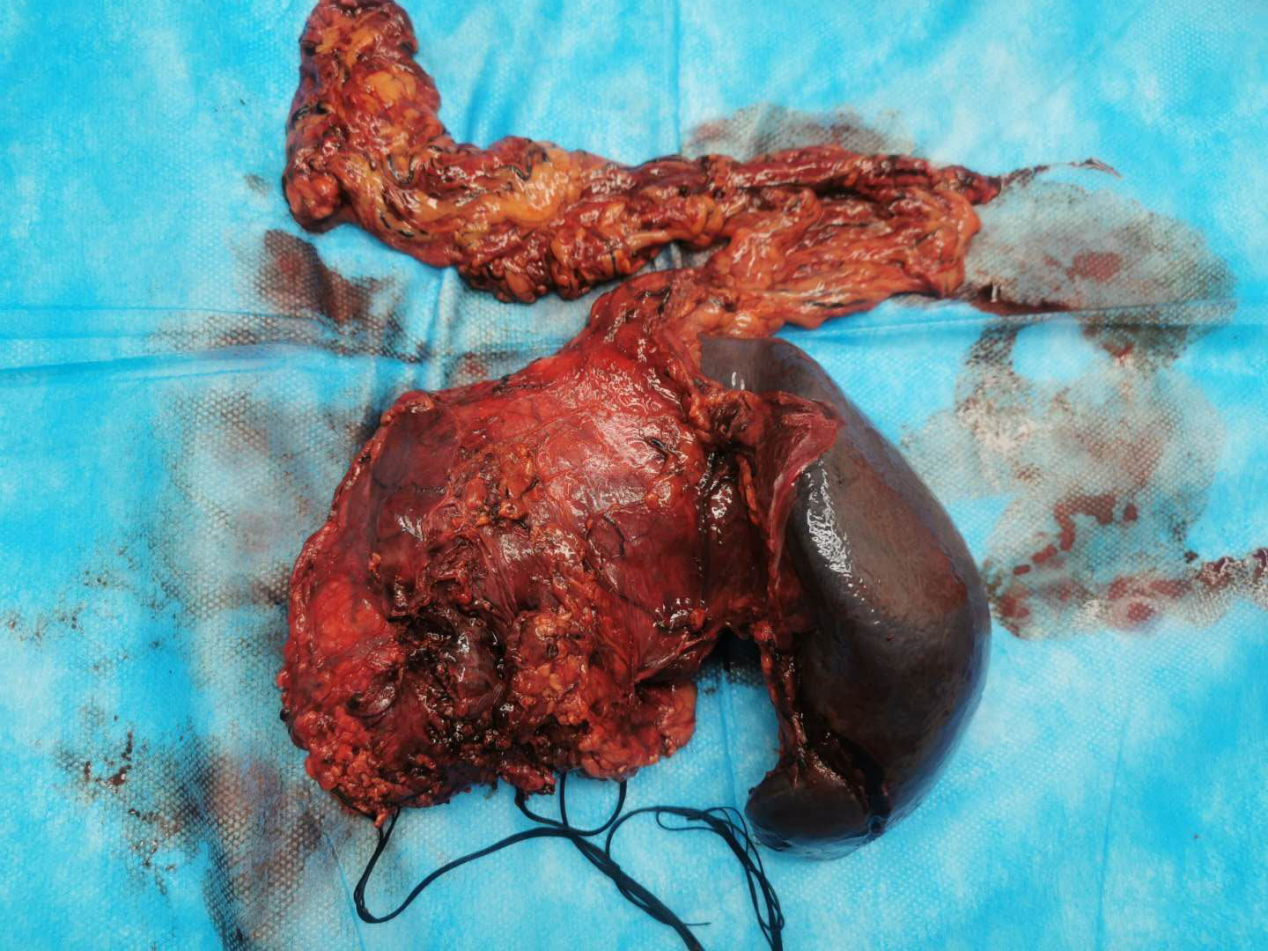
The mass was located in the body and tail of the pancreas, invading the spleen. The pancreatic tumor presented as soft and irregular.

**Figure 3:**
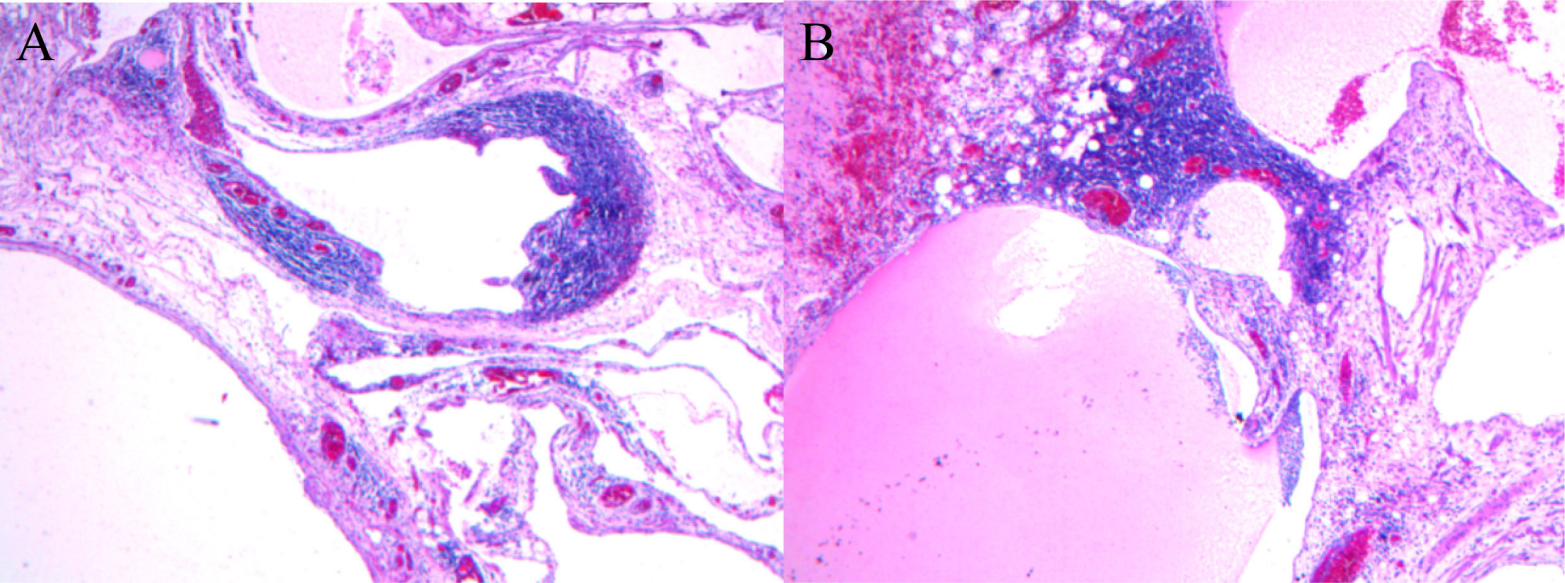
Histopathological microphotography (hematoxylin and eosin, ×100) revealed that the tumor was composed of lymphatic and blood vessels, featuring multiple cavities **(A)**, and contained jelly-like material **(B)**.

Due to the size of the tumor, the patient´s symptoms, and a high suspicion of malignancy, we informed the patient about his condition and presented several treatment options. After discussing these options, we received the patient’s treatment decisions. Consequently, we opted to perform an exploratory laparotomy. A multilocular cystic tumor was identified in the body and tail of the pancreas, invading the splenic artery and vein. Body and tail pancreatectomy along with spleen resection was performed (**Figure 2**). The total blood loss during the procedure was 400 ml, and the surgical time was 210 minutes. Histopathological analysis revealed a multilocular cyst in the pancreatic body and tail, measuring 12x10x3 cm, which contained both liquid and jelly-like material. The peripancreatic lymph nodes were normal. The final pathological diagnosis of this tumor was a hemolymphangioma of the pancreas (**Figure 3**). The results of the immunohistochemistry staining were as follows: CD31(+), CD34(+), D2-40(+), CKp(+), ER(-), PR(-), Ki67 (Lymphocytic active region 40%+).

The patient experienced a rapid recovery and was discharged 9 days post-surgery. Follow-up evaluations, which included ultrasonography at 3 months and CT at 1 year (**Figure 1D**) showed no signs of recurrence.

## 3 Discussion

Pancreatic hemolymphangioma is an uncommon, benign cystic tumor. A literature review conducted up to May 2024 (via an online PubMed search) found only 12 cases (**Table 1**). To summarize, pancreatic hemolymphangiomas are typically located at the head of the pancreas and primarily affect adult patients, with 84% (11/12) of cases involving individuals over the age of 25. This tumor is more prevalent in women, resulting in a male-to-female ratio of 1:5 (2/10) (7). The case discussed here involves the youngest male patient reported thus far, with the tumor located in the body and tail of the pancreas. Preoperative abdominal CT examination indicated signs of invasion into blood vessels. During surgical exploration, it was confirmed that the tumor invaded the splenic vein. As a result, it was initially presumed to be pancreatic cystadenocarcinoma during the operation, leading to the selection of body and tail pancreatectomy combined with total splenectomy. Postoperative pathology confirmed the diagnosis of pancreatic hemolymphangioma. Banchini et al. described this tumor as a congenital malformation of the vascular system (11). Nonetheless, two reports have noted the invasive characteristics of this tumor. Studies report that it has the ability to invade adjacent organs, such as the duodenum and the transverse mesocolon (14, 15). In this case, the tumor was invasive and giant. Additionally, this is the first instance of a “beaver tail” liver found in a patient with this tumor.

**Table 1:** Characteristics of 12 cases with hemolymphangioma of the pancreas.

Case number	Sex	Age (years)	Site of pancreas	Size (cm)	Treatment	Recurrence
1 (3)	F	68	Head	—	PD	No
2 (4)	F	66	Head	—	PD+PG	No
3 (5)	F	31	Body/tail	14	BTP	No
4 (6)	F	67	Head	15	PD	No
5 (7)	F	53	Head	4×3	PD	No
6 (8)	M	53	Head	6	PD	No
7 (9)	F	20	Head	18×16×12	PD	No
8 (10)	F	39	Body/tail	10×7	BTP	No
9 (11)	F	52	Head	8×6.5×6	PPPD	No
10 (12)	F	57	Neck/body	8×6×4.5	LR	No
11 (13)	M	28	Head	12×8.5×12	PPPD	No
12 (15)	F	30	Body/tail	12×10×7.5	BTP+ middle colic artery and vein resection were performed	No

NA, not available; PD, pancreatoduodenectomy; PG, partial gastrectomy; BTP, body and tail pancreatectomy; LR, local resection.

Typically, preoperative abdominal contrast-enhanced CT scans display multilocular low-density heterogeneous masses, with enhancement of cyst septum after contrast administration. An abdominal MRI is recommended for a definitive diagnosis. MRI images showed both cystic and solid components (7). The T1-weighted (T1W1) images primarily exhibited low signals, while the T2-weighted (T2W1) images showed predominantly high signals (2). Tumor markers remained within normal ranges in laboratory tests. There was insufficient evidence to definitively classify the tumor as cystadenoma, cystadenocarcinoma, or other relatively common cystic-solid pancreatic tumors; hence, pancreatic hemolymphangiomas should be included among the differential diagnoses. Unfortunately, the patient did not undergo an abdominal MRI prior to surgery, which may have provided more insight and potentially benefited him from a multi-disciplinary treatment model standpoint. Hemolymphangiomas typically present as cystic or cavernous lesions. Microscopically, the tumor consists of abnormal lymphatic and blood vessels with polycystic spaces. Immunohistochemistry can aid in further distinguishing this tumor: both vascular and lymphatic endothelial cells express CD31 and CD34, whereas D2-40 is expressed only in lymphangioma and some malignant vascular tumors (14).

Although postoperative recovery is generally favorable and the risk of recurrence and metastasis is low, long-term regular follow-up is recommended (2, 7). Abdominal ultrasound is cost-effective and often the first choice for examination, while abdominal CT or MRI can serve as alternatives. In this case, follow-up via ultrasonography and CT (**Figure 1D**) revealed no signs of recurrence.

## 4 Conclusion

Despite the low incidence of this tumor, comprehensive preoperative imaging examination and careful interpretation of imaging data are essential for accurate diagnosis. Active surgical treatment remains the best option for this condition and long-term follow-up is crucial.

## Data Availability

The original contributions presented in the study are included in the article/Supplementary Material. Further inquiries can be directed to the corresponding author.
